# Robotic liver resection in minimally invasive expert centers is associated with a very low rate of bile leakage: results from a national multicenter cohort

**DOI:** 10.1007/s11701-026-03507-1

**Published:** 2026-05-27

**Authors:** Víctor López-López, Jordi Navinés-López, Gonzalo Gómez, Dilmurodjon Eshmuminov, Marta García-Sánchez, Isabel Jiménez, Ignacio Sánchez-Esquer, Cecilia Maina, Javier Briceño, Esteban Cugat, Ricardo Robles-Campos

**Affiliations:** 1 Department of General, Visceral and Transplantation Surgery, Clinic and University Hospital Virgen de La Arrixaca, IMIB-Pascual Parrilla. Murcia University, Ctra. Madrid-Cartagena, s/n, El Palmar, 30120 Murcia, Spain; 2 Department of General Surgery, Hospital Universitari General de Catalunya, Sant Cugat, Barcelona Spain; 3 Department of General Surgery and Transplantation, Hospital Universitario Reina Sofía, Córdoba, Spain; 4 Department of Surgery and Transplantation, University Hospital Zurich, Zurich, Switzerland; 5 Emergency Medical Center, Virgen de la Victoria University Clinical Hospital, Malaga, Spain; 6 Hepatobiliary Surgery Division, IRCCS San Raffaele, Milan, Italy; 7 Unidad de cirugía HPB Hospital Universitario Germans Trias y Puyol, Universidad Autónoma de Barcelona, Barcelona, Spain

**Keywords:** Robotic liver surgery, Biliary leaks, Expert centers, Postoperative complications

## Abstract

Bile leakage remains one of the most relevant complications after liver resection, contributing substantially to postoperative morbidity. Although minimally invasive liver surgery has reduced biliary complications compared with open approaches, robust multicenter data specifically evaluating bile leakage after robotic liver resection in expert centers remain limited. To assess bile leak incidence and severity following robotic liver resection in expert centers. This national, multicenter observational database included all consecutive adult patients undergoing robotic liver resection in three high-volume reference centers. Patients requiring biliary diversion or living donor procedures were excluded. Perioperative outcomes were prospectively collected, focusing on postoperative bile leakage, defined and graded according to the International Study Group of Liver Surgery (ISGLS) criteria. Secondary outcomes included overall morbidity, conversion rate, mortality, and length of hospital stay. A total of 492 patients were included. Malignant disease was the primary indication in 80% of cases, and 62% of resections were anatomical. Conversion to open or laparoscopic surgery occurred in 2.6%. Postoperative bile leakage occurred in 9 patients (1.8%). According to ISGLS classification, 6 patients (67%) developed Grade A bile leaks and 3 patients (33%) Grade B leaks; no Grade C bile leaks were observed. All biliary complications were managed conservatively or with minimally invasive interventions, without the need for surgical reintervention. Overall postoperative morbidity within 90 days was 29.3%, with a 90-day mortality of 1.6%. Median length of stay was 3 days (IQR 3–5). In this national multicenter cohort from expert centers, robotic liver resection was associated with a very low incidence of bile leakage and an absence of severe (Grade C) biliary complications. These findings support the safety of robotic liver surgery and highlight the key role of high-volume centralization in keeping biliary outcomes.

## Introduction

Liver surgery has evolved significantly over the past few decades, standing on the minimally invasive surgery (MIS) background as an alternative to traditional open techniques [1–4]. Robotic surgery has emerged as a safe and viable option, offering advantages in terms of precision, control, and visualization [5–7]. However, despite its benefits, robotic liver surgery (RLS) remains an area of study and debate, particularly regarding its impact on postoperative complications, especially biliary leaks (BL), one of the most frequent and challenging complications following liver resections.

Biliary leaks are a significant complication that affects postoperative morbidity, and their incidence varies depending on various factors, including the surgical technique, the surgeon’s experience, and the complexity of the procedure [8–11]. Laparoscopic surgery has been shown to reduce the incidence of biliary leaks compared to open surgery, but the impact of robotic surgery on this complication remains under investigation [12]. The main advantage of robotic surgery lies in the ability to perform liver resections with greater precision, enhanced three-dimensional visualization, ease of intracorporeal suturing, and delicate vital structure handling, factors that may potentially reduce the risk of BL [13].

Several studies have reported promising results with RLS, particularly when performed by experienced surgeons in high-volume centers [14–17]. However, the experience and outcomes from centers with extensive experience in robotic liver surgery remain limited. This study focuses on the incidence of biliary leaks associated with robotic approach in liver surgery in groups of surgeons with experience in minimally invasive surgery. This analysis could contribute to establishing a reference framework regarding the safety and efficacy of the robotic approach in liver surgery, promoting its adoption and optimization in clinical practice.

## Materials and methods

### Study design

This is a multicenter study conducted across the three high-volume centers with established robotic liver surgery programs. Data were prospectively collected from patients undergoing RLS for liver resection between April 23, 2018, and December 30, 2024. The study design allowed for the continuous and systematic collection of preoperative, intraoperative, and postoperative data. A primary focus was placed on analyzing the incidence of biliary leaks.

The institutional review board at the Clinic and University Virgen de la Arrixaca Hospital (Murcia, Spain) approved the study (Internal Protocol Code: 2026-2-4-HCUVA). The study adhered to the principles of the Declaration of Helsinki, and patient confidentiality was maintained throughout the research process. All data were anonymized, and any identifying information was removed to protect patient privacy.

### Inclusion and exclusion criteria

Patients included in the study were aged 18 years or older and underwent RLS. Eligible patients had a clear surgical indication as determined by preoperative imaging, clinical assessment, and multidisciplinary team evaluation. The study included patients with both primary or secondary liver diseases requiring resection. All patients were deemed suitable candidates for robotic surgery by the attending surgeon based on preoperative evaluations, which were reviewed and approved by a multidisciplinary committee. Furthermore, patients were required to have adequate follow-up post-surgery to ensure complete outcome data could be collected.

Exclusion criteria were carefully defined to ensure homogeneity in the study population. Patients who required biliodigestive diversion (that is, including biliary duct resection) or living donors were excluded due to these procedures represent distinct surgical scenarios with substantially different biliary risk profiles. Anatomical factors that made robotic assistance infeasible, such as extensive adhesions or advanced cirrhosis, were also considered as exclusion criteria. Finally, patients with incomplete clinical data or insufficient postoperative follow-up were excluded from the analysis.

### Surgical technique

Robotic liver resections were performed using the da Vinci Xi or X robotic platforms. Patient positioning and trocar placement followed center-specific standardized configurations, typically consisting of four robotic ports and one assistant port adapted to lesion location and surgeon preference. Intraoperative ultrasound was routinely used to confirm tumor location and guide the transection plane. Liver parenchymal transection during robotic liver surgery was performed predominantly using microfracture techniques with robotic scissors and Kellyclasia using the robotic Maryland dissector. Energy devices, including SynchroSeal and Vessel Sealer, were mainly used during major hepatectomies or in cases requiring larger parenchymal transection planes, according to surgeon preference and intraoperative requirements. All procedures were completed using a fully robotic technique. The use of indocyanine green (ICG) fluorescence imaging or intraoperative bile leak testing was applied selectively according to institutional protocols. Conversion to laparoscopic or open surgery was undertaken in cases of uncontrolled bleeding, inability to safely progress with robotic dissection, or other intraoperative safety concerns. All procedures were carried out by surgeons experienced in minimally invasive liver surgery within high-volume hepatobiliary centers, each with established RLS programs and center-specific technical adaptations.

### Data collection and variables

Data were collected prospectively from clinical databases at the participating centers. A comprehensive dataset was compiled including demographic, clinical, surgical, and postoperative information. Demographic variables included patient age, sex, body mass index (BMI), and the American Society of Anesthesiologists (ASA) score. Clinical factors, such as comorbidities (e.g., diabetes mellitus, hypertension), history of previous abdominal surgery, and liver functionlabs, were also collected. Preoperative imaging studies, including contrast-enhanced CT scans or MRI, were used to assess the extent of liver lesions and anatomical considerations for surgery.

Intraoperative data included the type of surgical procedure and the duration of the surgery. Intraoperative complications, such as bleeding or need for conversion were also noted. Postoperative data collection focuses on complications, particularly biliary leaks. The severity of biliary leaks was classified according to the International Study Group of Liver Surgery (ISGLS) grading system: Grade A (mild), Grade B (moderate), and Grade C (severe). Other complications, including surgical site infections (SSI), hemorrhage, and postoperative liver failure, were also documented. The need for additional interventions or prolonged hospital stays was recorded as part of the postoperative outcome measures.

### Statistical analysis

Descriptive statistics were used to summarize the demographic and clinical characteristics of the patient cohort. Continuous variables, such as age, BMI, and surgery duration, were expressed as means ± standard deviations, while categorical variables (e.g., sex, comorbidities, complication rates) were summarized as frequencies and percentages. The main objective of the study was to analyze the incidence of biliary leaks following RLS. To assess potential risk factors for biliary leaks, subgroups were analyzed based on surgical technique, surgeon experience, and the presence of preexisting liver risk factors. A significance level of *p* < 0.05 was considered statistically significant.

## Results

### Baseline characteristics

A total of 492 patients were included in the study, with a median age of 64 years (IQR 56–73). The cohort comprised 52% male and 48% female patients. The median BMI was 31 (IQR 26–45.7), and most patients had an ASA score of II (45%) or III (48%). A significant proportion (35%) had a history of previous abdominal surgery, with the majority having undergone laparoscopic procedures (48.3%), followed by open surgery (42.5%) and robotic surgery (6.5%) (Table 1).

**Fig. 1:**
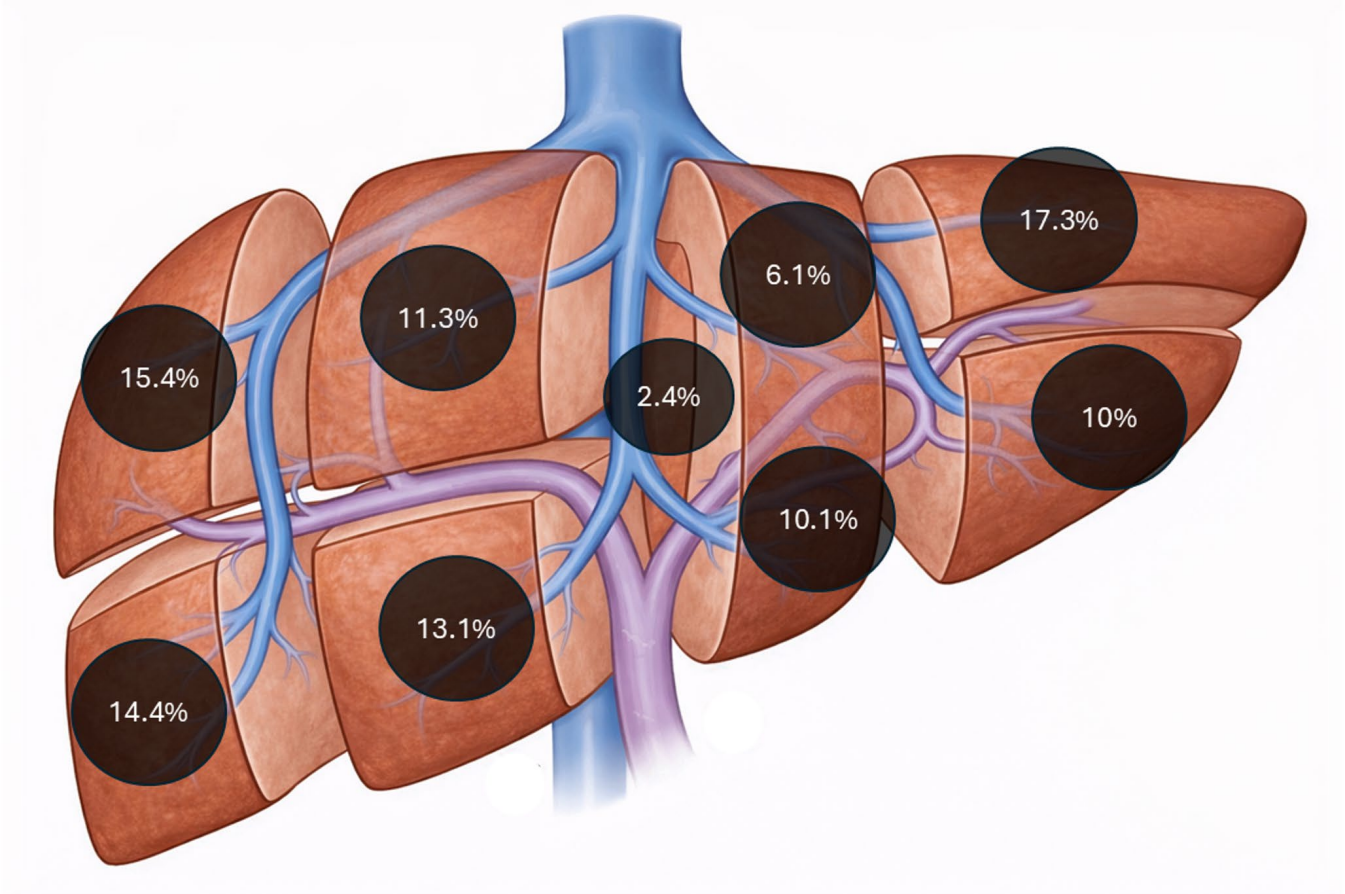
Distribution of resected liver lesions according to Couinaud segments. Schematic representation of the anatomical distribution of liver lesions included in the study cohort

Regarding the primary diagnosis, 80% of patients had malignant lesions, with the most common being colorectal liver metastasis (38%), hepatocellular carcinoma (22%), and cholangiocarcinoma (8.3%). Most patients (80%) had a single lesion, and the median lesion size was 3 cm (IQR 1.8–5.2). Lesions were predominantly located in segments 2, 3, 5, 6, and 7 of the liver, with 18.1% of patients having lesions near major vessels (Fig. 1). Neoadjuvant chemotherapy was administered to 34% of patients.

**Table 1:** Baseline Characteristics

Characteristic	*N* = 492^1^
**Age**,** years**,** median (IQR)**	64 (56, 73)
**Female**	234 (48%)
**BMI**,** kg/m²**,** median (IQR)**	31 (26, 45,676)
**ASA**	
I	23 (4.7%)
II	220 (45%)
III	235 (48%)
IV	14 (2.8%)
**Previous abdominal surgery**	174 (35%)
**Previous surgery type**	
Open	74 (42.5%)
Laparoscopic	84 (48.3%)
Robotic	11 (6.5%)
NA	5 (2.9%)
**Liver disease**	
HBV	13 (2.6%)
HCV	30 (6.1%)
Alcohol	33 (6.7%)
MAFLD	11 (2.2%)
PSC	1 (0.2%)
**Diagnosis type**	
Malignant	393 (80%)
Benign	99 (20%)
**Malignant cause**	
CRLM	187 (38%)
HCC	108 (22%)
ICC	41 (8.3%)
GBC	16 (3.3%)
Non-CRLM	39 (7.9%)
Other	2 (0.4%)
**Benign type**	
Hemangioma	18 (3.7%)
Adenoma	23 (4.7%)
FNH	10 (2%)
Cysts	35 (7.1%)
Others	13 (2.6%)
**Lesion size**,** median, cm (IQR)**	3 (1.8, 5.2)
**Vessel proximity**	89 (18.1%)
**Neoadyuvant chemotherapy**	165 (34%)

¹n (%). IQR, interquartile range; BMI, body mass index; ASA, American Society of Anesthesiologists physical status classification; NA, not available; HBV, hepatitis B virus; HCV, hepatitis C virus; MAFLD, metabolic dysfunction-associated fatty liver disease; PSC, primary sclerosing cholangitis; CRLM, colorectal liver metastases; HCC, hepatocellular carcinoma; ICC, intrahepatic cholangiocarcinoma; GBC, gallbladder cancer; FNH, focal nodular hyperplasia; S1-S8, Couinaud liver segments 1–8

**Table 2:** Intraoperative Data

Operative Outcome	*N* = 492^1^
**Resection type**	
Anatomical	302 (62%)
Non-anatomical	166 (34%)
Combination	24 (4.9%)
**Type of Hepatectomy**	
Minor	431 (88%)
Major	61 (12%)
**Type of resection**	
Wedge resection	158 (32.1%)
Segmentectomy	142 (28.9%)
Left lateral sectionectomy	93 (18.9%)
Left hepatectomy	31 (6.3%)
Right hepatectomy	29 (5.9%)
Right posterior sectionectomy	6 (1.2%)
Others	32 (6.5%)
**IWATE**,** median (IQR)**	6 (4, 8)
**Operative time**,** min**,** median (IQR)**	209 (150, 270)
**Conversion**	13 (2.6%)
Laparoscopic	2 (0.4%)
Open	11 (2.2%)
**Conversion reason**	
Bleeding	5 (1%)
Technical challenge	6 (1.2%)
Planned	2 (0.4%)
**Pringle**	405 (82.3%)
**Pringle time**,** min**,** median (IQR)**	35 (19, 54)
**Blood loss**,** ml**,** median (IQR)**	150 (100, 300)
**Transfusion**	19 (3.9%)
**Blood_Units**,** median (IQR)**	1 (1,2)

n (%). IQR, interquartile range; IWATE, Iwate difficulty score; min: minutes; ml: milliliters

**Table 3:** Postoperative Outcomes

Outcome	*N* = 492^1^
**Overall complications at 90days**	144 (29.3%)
**Clavien-Dindo**	
1	63 (12.8%)
2	40 (8.1%)
3a	19 (3.9%)
3b	7 (1.4%)
4a	6 (1.2%)
4b	1 (0.2%)
**Mortality at 90 days**	8 (1.6%)
**CCI**, median (IQR)	0 (0, 9)
**PHLF**	14 (2.8%)
A	7 (1.4%)
B	3 (0.6%)
C	4 (0.8%)
**Hemorrhage**	9 (1.8%)
**Abdominal collection**	32 (6.5%)
**Surgical site infection**	18 (3.7%)
**Radicality**	
R1	71 (14.4%)
**Hospital stay**,** days** median (IQR)	3 (3, 5)
**Readmission**	36 (7.3%)

¹n (%). IQR, interquartile range; PHLF, post-hepatectomy liver failure; R0, microscopically margin-negative resection; R1, microscopically margin-positive resection

**Table 4:** Postoperative Biliary Leak Subgroup

Variable	*N* = 9¹
**Age**,** years**,** median (IQR)**	59 (55–64)
**Female**	5 (55.6%)
**BMI**,** kg/m²**,** median (IQR)**	24.8 (22.5–28.2)
**Malignant etiology**	6 (66.7%)
**Neoadjuvant chemotherapy**	3 (33.3%)
**Number of lesions median (IQR)**	1 (1–1)
**Lesion size**,** cm**,** median (IQR)**	1.7 (1.5–7.6)
**Resection type**	
Anatomical, n (%)	7 (77.8%)
Non-anatomical, n (%)	1 (11.1%)
Combination, n (%)	1 (11.1%)
**Operative time**,** min**,** median (IQR)**	257.5 (166.8–303.8)
**Pringle maneuver**	8 (88.9%)
**Pringle time**,** min**,** median (IQR)**	20 (10–35)
**Length of stay**,** days**,** median (IQR)**	6 (3–11)
**Comprehensive Complication Index (CCI)**	20.9 (20.9–26.2)
**Biliary leak (ISGLS)**	
Grade A	6 (66.7%)
Grade B	3 (33.3%)
Grade C	0

¹n (%). BMI, body mass index; CCI, Comprehensive Complication Index; IQR, interquartile range; ISGLS, International Study Group of Liver Surgery

### Intraoperative outcomes

Cirrhosis was present in 18% of patients. 62% of the surgeries were anatomical resections (Table 2). The cohort included predominantly parenchymal-sparing and minor resections, including wedge resections (32.1%), segmentectomies (28.9%), and left lateral sectionectomies (18.9%). Major hepatectomies accounted for 12.4% of the series, including right hepatectomy (5.9%) and left hepatectomy (6.3%). The median operative time was 209 min (IQR 150–270), with 2.6% (*n* = 13) of surgeries requiring conversion. Of those, 11 were converted to open surgery, and 2 to laparoscopic surgery. The main reasons for conversion included bleeding and technical non-progress.

Regarding intraoperative management, Pringle maneuver was used in 82.3% of surgeries, with a median Pringle time of 35 min (IQR 19–54). The median blood loss was 150 ml (IQR 100–300), with 3.9% of patients requiring blood transfusions. The majority of patients did not need blood units, but a small proportion required one or more units (2.8%).

### Postoperative outcomes

Complications occurred in 29.3% of patients within 90 days, and the 90-day mortality rate was 1.6%. The Clavien-Dindo classification showed 12.8% had minor complications (Grade 1), and 8.1% had moderate complications (Grade 2). Severe complications (≥ 3a) were 8.3%. Postoperative liver failure (PHLF) occurred in 14 patients (2.8%), with the majority classified as grade A. Hemorrhage also occurred in 1.8% of patients. Finally, abdominal collections and surgical site infections were observed in 6.5% and 3.7% of patients, respectively. Oncologically, 85.6% of patients underwent R0 resections. The median length of stay (LOS) was 3 days (IQR 3–5), and 7.3% of patients required readmission within 90 days (Table 3).

### Biliary complications cohort

Among the 9 patients (1.8%) who developed postoperative biliary leakage, the median age was 59 years, 5 patients were female and BMI was 24.8 kg/m². Regarding lesion etiology, 6 had a malignant etiology and 3 patients (33%) had received neoadjuvant chemotherapy prior to surgery (Table 4).

Tumor characteristics showed an overall limited disease burden, with a median number of lesions of 1, and lesion size in the low-centimeter range when available. Anatomical resections were more frequent (*n* = 7) among patients who developed biliary leakage. The median operative time was prolonged (257.5 min) and Pringle maneuver was used in 8 of the 9 patients (89%), with a median clamping time of 20 min.

Regarding the outcomes, the median length of stay was 6 days. Overall morbidity remained limited, with a median Comprehensive Complication Index (CCI) of 20.9. According to the International Study Group of Liver Surgery (ISGLS) classification, biliary leakage was predominantly mild, with 6 patients (67%) presenting Grade A leaks and 3 patients (33%) Grade B leaks. Interestingly, no Grade C biliary leaks were observed, and all cases were managed conservatively or with minimally invasive measures, with no need for surgical reintervention.

## Discussion

This multicenter study represents the most comprehensive national experience of robotic liver surgery performed in expert centers in Spain. It shows excellent perioperative outcomes, even under a high degree of technical complexity. Overall postoperative morbidity was low, with particularly favorable rates of biliary leaks, bleeding, intra-abdominal collections, and surgical site infections, along with a very limited need for conversion. These results improve the degree of evidence of the robotic approach in liver surgery as a safe and effective approach when performed in specialized centers with consolidated experience in minimally invasive techniques.

The most clinically relevant and distinctive finding of this study is the very low incidence and limited severity of biliary complications. Bile leaks are a common complication after liver resection, with reported incidence rates typically ranging from 2.6% to 27.2%, depending on surgical complexity [9, 18–20]. While some series have described rates as low as 2.6–7.6% in selected cases, higher-complexity procedures (particularly major hepatectomies) are frequently associated with bile leak rates in the range of 10–15%. In contrast, the incidence of biliary leakage in this robotic series was remarkably low and, importantly, all events were classified as low-grade according to the ISGLS classification. It should be noted that the median operative time was prolonged, reflecting a high surgical complexity in these series.

Of particular relevance is the complete absence of Grade C biliary leaks in this cohort. Grade C biliary leakage, which requires surgical reintervention, represents the most severe form of this complication and is associated with substantial morbidity, prolonged hospitalization, and increased healthcare costs. The absence of Grade C leaks in this study, despite the complexity of the resections performed, strongly reinforces the concept that RLS is associated not only with a low incidence of biliary leakage but also with a minimal clinical impact when leakage occurs.

An important consideration is that the participating expert centers routinely apply a selective rather than systematic drainage policy after robotic liver resection. Although this may theoretically reduce detection of asymptomatic low-grade leaks, postoperative biliary complications were rigorously assessed through imaging, clinical follow-up, and analysis of drained collections when required. Importantly, intra-abdominal collections were differentiated from bilomas, as no biliary content was identified in drained fluid samples.

Several factors may explain these favorable biliary outcomes. First, all procedures were performed in high-volume centers with extensive experience in minimally invasive and robotic liver surgery. Second, the robotic platform enhances the three-dimensional visualization of biliary anatomy, tremor filtration, and precision for fine dissection and intracorporeal suturing, which are critical elements in preventing and managing biliary injuries. Finally, standardized surgical strategies and careful inspection of the transection surface likely contributed to early identification and control of minor bile leaks before they evolved into clinically significant complications.

The low rate of conversion observed in this series deserves special attention. Conversion rates in liver surgery are widely recognized as an indirect marker of intraoperative difficulty and surgical safety [2, 21, 22]. In previously published series of laparoscopic liver surgery, conversion rates ranging from 5% to 15% have been reported, particularly in complex resections, cirrhosis, posterior segments, or cases requiring vascular control [23, 24]. In contrast, the conversion rate observed in the present robotic series was markedly lower, despite the inclusion of anatomically complex resections and lesions located in technically demanding areas. This finding highlights the added value of the robotic platform in maintaining minimally invasive access even in challenging scenarios, likely due to enhanced dexterity, stable instrumentation, and superior visualization.

Postoperative bleeding and intra-abdominal collections, two of the most frequent complications after liver resection, were also infrequent in this cohort. Open liver surgery has traditionally been associated with higher rates of blood loss and transfusion, while laparoscopic approaches have improved these outcomes but still present limitations in complex cases [25–27]. The low incidence of hemorrhagic complications observed in this study suggests that robotic assistance allows for precise parenchymal transection and effective vascular control, even when Pringle maneuver or vascular resections are required. Similarly, the low rate of intra-abdominal collections supports meticulous management of the transection surface and careful intraoperative technique [13].

Margin status remains a major challenge in highly complex liver surgery requiring vascular proximity or reconstruction. Notably, 26.8% of R1 resections corresponded to vascular R1 resections related to tumor proximity to major vascular structures, most commonly in colorectal liver metastases. In these cases, vascular detachment was intentionally performed to preserve critical inflow or outflow structures and avoid excessively extended resections. As previously reported in the hepatobiliary literature, particularly for colorectal liver metastases, vascular R1 resections may achieve oncological outcomes comparable to R0 resections in carefully selected patients.

Taken together, these results strongly support the central message of this study that RLS performed in expert centers is associated with low overall morbidity and an exceptionally low severity of biliary complications. Compared with open and laparoscopic approaches, the robotic platform appears to offer tangible advantages in complex liver surgery, particularly in reducing the incidence and clinical consequences of biliary leakage and improving quality of life of the patients [28].

This study has several limitations that should be acknowledged. Although data collection was prospective, the analysis remains observational and does not include a direct comparison with open or laparoscopic liver surgery. Consequently, comparisons with other surgical approaches rely on previously published series rather than an internal control group. Nevertheless, the primary objective of the study was to evaluate the outcomes of robotic liver surgery in expert centers, with particular emphasis on biliary complications. All participating institutions were high-volume hepatobiliary centers with extensive experience in minimally invasive liver surgery. While this likely contributed to the favorable outcomes observed, it may limit the generalizability of the findings to lower-volume centers or surgeons earlier in the learning curve. At the same time, the multicenter design provides a broad representation of current expert practice across different institutions and patient populations. Finally, detailed histopathological characterization of chemotherapy-associated liver injury, including sinusoidal injury and steatohepatitis, was not systematically available across centers and therefore could not be specifically analyzed as a risk factor for postoperative bile leak. However, metabolic dysfunction-associated steatotic liver disease was identified in only 2.2% of patients, and 33.5% of the cohort had received preoperative chemotherapy, predominantly FOLFOX-based regimens.

In this large national multicenter experience from expert centers, RLS was associated with low overall morbidity, minimal conversion rates, and excellent control of traditionally feared complications such as bleeding and intra-abdominal collections. Most importantly, biliary leakage was infrequent and uniformly low-grade, with no Grade C biliary leaks observed. These findings support the concept that robotic technology, when combined with surgical expertise, significantly may contribute to reduces the clinical impact of biliary complications, positioning robotic liver surgery as a safe and highly effective approach for complex hepatic resections.

## Data Availability

Data is provided within the manuscript.
